# The Trend of Risk for Cardiovascular Diseases During the Past Decade in Iran, Applying No-Lab and Lab-Based Prediction Models

**DOI:** 10.5334/gh.1180

**Published:** 2023-02-10

**Authors:** Noushin Fahimfar, Karim Kohansal, Samaneh Asgari, Afshin Ostovar, Farzad Hadaegh, Davood Khalili

**Affiliations:** 1 Osteoporosis Research Center, Endocrinology and Metabolism Clinical Sciences Institute, Tehran University of Medical Sciences, Tehran, Iran; 2 Department of Epidemiology and Biostatistics, School of Public Health, Tehran University of Medical Sciences, Tehran, Iran; 3 Prevention of Metabolic Disorders Research Center, Research Institute for Endocrine Sciences, Shahid Beheshti University of Medical Sciences, Tehran, Iran; 4 Center for Non-communicable Diseases, Ministry of Health, Tehran, Iran

**Keywords:** Cardiovascular disease, primary prevention, risk assessment, prediction models, Diabetes

## Abstract

**Background:**

As a surrogate for all relevant risk factors, it is preferable to show trends in the mean cardiovascular disease(CVD) risk rather than to examine each risk factor trend separately.

**Objective(s):**

Using national representative data, this study aimed to determine the changes in the World Health Organization (WHO) CVD risk during the last decade considering both laboratory and non-laboratory risk scoring.

**Methods:**

We used data from five rounds of the WHO STEPwise approach to surveillance surveys (2007–2016). In all, 62,076 (31,660 women) participants aged 40–65 years were included and their absolute CVD risk were calculated. The generalized linear model was performed to assess the trend of CVD risk in men and women, and also in diabetic and non-diabetic individuals.

**Results:**

We showed significant declining trends in the mean CVD risk in the laboratory (from 10.5% to 8.8%) and non-laboratory (10.1% to 9.4%) models in men. In women, a significant reduction was observed in the laboratory-based model (from 8.4% to 7.8%). The laboratory model showed a greater decrease in men than women (P-for interaction < 0.001) and in diabetic patients (from 16.1% to 13.6%) than non-diabetic individuals (from 8.2% to 7%) (p-for interaction = 0.002). The proportion of high-risk individuals (risk ≥ 10%) decreased from 40% in 2007 to 31.5% in 2016 in men and from 29.8% to 26.1% in women based on the laboratory-model.

**Conclusions:**

During the last decade, CVD risk had a significant decrease in men and women. The reduction was more evident in men and diabetic population. However, still, one-third of our population is considered high-risk.

## Introduction

Cardiovascular diseases, as the main cause of death in Iran, accounts for 43% of total mortalities [1]. Risk factors of cardiovascular diseases (CVDs), including diabetes, hypertension, and obesity, are prevalent and need urgent prevention programs. Screening high-risk individuals using the clinical prediction models is crucial to assign them to prevention programs [2]. Showing this calculated risk as a surrogate of all related risk factors together is preferred to looking at each risk factor’s trend separately [3,4].

Having information on the trend of CVD risk and related risk factors at the population level would provide insights regarding the possibility of achieving the WHO’s goals. This approach aimed to reduce the burden of chronic diseases worldwide, including a 25% relative reduction in premature mortality, mainly caused by CVD, and targets major risk factors of CVDs [5].

It seems that the trend of CVD risk factors has not followed a similar pattern during the last decade in Iran. A study on people aged ≥60 years in the Tehran Lipid and Glucose Study (TLGS) showed that the prevalence of diabetes, hypertension, and central obesity had a noticeable upward trend during 2002–2014; however, lipid profile parameters showed favorable trends [6]. In the Isfahan Cohort study, from 2001 to 2013, the prevalence of metabolic syndrome, hypertension, abdominal obesity, and dysglycemia (diabetes and Impaired Glucose Tolerance) increased over the 12 years, while the prevalence of hypertriglyceridemia decreased during this period [7]. Achieving and monitoring the targets require a robust national surveillance system. For this reason, the WHO STEPwise approach to surveillance (STEPS) was recommended as a part of the surveillance program for the non-communicable disease risk factors. The STEPS approach helps countries strengthen their capacity by obtaining core data on the risk factors leading to major disease burdens. Fortunately, STEPS studies in Iran have prepared such a system. According to these studies, it has been shown that the prevalence of metabolic syndrome among adults aged 25–64 years was 35.9% (95% CI, 34.3–37.6%) in 2007, which decreased to 33.0% (30.7–35.2%) in 2011 (P = 0.011); however, the prevalence of central obesity, high triglycerides, and low HDL-cholesterol remained constant and the prevalence of increased fasting plasma glucose increased [8].

It seems that showing the trend of CVD risk, as a surrogate of all related risk factors, is preferred to look at each risk factor’s trend separately. On the other hand, national data about incident cardiovascular disease is so challenging to collect; thus, an alternative source of information is needed to generate insights into this important surveillance need. Using the recent well-known prediction models including the laboratory and non-laboratory WHO model [9], the current study aimed to estimate the trend of 10-year CVD risk scoring during the recent years in a nationally representative population of Iran.

## Methods

We used data available from five national STEPs surveys (2007, 2008, 2009, 2011, and 2016). Due to some variations in the measurement methods, the data of STEPS 2005 was not included in this study. All studies were conducted using the standardized approaches introduced by the World Health Organization (WHO), and all experimental protocols were approved by the Center for Disease Control, Ministry of Health and Medical Education in Iran. Informed consent was received from all participants. A short description of the sampling methods is presented as follows.

### Study population

In all, 72,128 population aged 25–65 years were included in the STEPS (2007: 23,487, 2008: 23,290, 2009: 23,334, 2011: 7,551, 2016: 23,738). Although some differences were found in studies, using random cluster sampling methods, all surveys provided representative samples of the Iranian population. A randomized cluster sampling at the levels of towns, villages, and districts was used in 2007, 2008, and 2009 including 1,000 individuals in each province.

A multistage cluster random sampling was used in 2011. Fifty primary sampling units (PSUs) were selected from the list of PSUs of distinct counties. After that, twelve secondary sampling units (SSUs) were chosen from the SSUs that were listed from urban and rural areas. Then, 20 postal addresses were randomly selected in each SSU. Finally, using Kish tables provided by WHO, one person was selected and participated from each household.

A systematic cluster random sampling was used in 2016 in which 3,105 clusters (10 subjects from each cluster) were selected from urban and rural areas of all provinces. The minimum sample size was estimated based on the province with the lowest population density. In provinces with more than 800 clusters, to minimize the estimated costs, half of the required sample size was considered, applying the doubled weight in the analysis.

Since the CVD prediction models mostly targeted the population aged over 40 years, age ranges between 40 to 65 years were considered for this study. Implausible data such as systolic blood pressure (SBP) less than 70 mm Hg or more than 270 mm Hg, body mass index (BMI) more than 80 kg/m2, and total cholesterol less than 70 mg/dl and more than 770 mg/dl were excluded.

### Measurements

All required information including demographic characteristics, past medical history of diseases, medication use, and behaviors such as smoking and physical activity were collected using standard questionnaires based on the WHO STEPS. The WHO STEPwise approach to non-communicable disease risk factor surveillance (STEPS) is a simple and standardized method for collecting, analyzing, and disseminating data on key NCD risk factors in countries. These kinds of surveys are repeated over time to monitor the situation of countries (World Health Organization. WHO STEPS surveillance manual: the WHO STEPwise approach to chronic disease risk factor surveillance. World Health Organization; 2005.) Blood pressure was measured using the appropriate cuff sizes sphygmomanometers. Clinical examination was conducted using a standard protocol and all staff was trained before the study. According to a standard protocol, laboratory measurements were assessed after 12–14 h overnight fasting. Among all surveys, laboratory measurements were collected in 2007, 2011, and 2016. All steps were supervised through periodic monitoring by different levels and reported to higher-level officials. Verbal informed consent was attained from each participant before the study enrollment.

### Definition of terms

The average of two measurements of blood pressure was considered as the participant’s blood pressure. Diabetes was defined as a history of taking diabetes medication or Fasting Plasma Glucose ≥ 126 mg/dl. Smoking was defined as the current use of tobacco products. Body mass index (BMI) was calculated as the weight in kilograms divided by the height in meters squared.

### CVD risk scoring

The 10-year risk for cardiovascular diseases including myocardial infarction, fatal coronary heart disease, or stroke event was estimated using the variables and parameters reported for the laboratory and non-laboratory models [9]. WHO risk models were driven from individual data from many prospective cohort studies. The external validity of the models was assessed using the prospective cohort studies that did not contribute to the model development. The Tehran Lipids and Glucose Study met all criteria and was used as one of the cohorts in the external validation stage. The laboratory model estimates the 10-year risk of CVD for individuals using their age, systolic blood pressure, diagnosis of diabetes, smoking status, and total cholesterol. In the non-laboratory model diabetes and total cholesterol are replaced by BMI. The absolute CVD risk is calculated for men and women using different statistical models [9]. We categorized the CVD risk as <10%, 10 – <20%, 20 – <30% and ≥30%.

### Statistical analysis

Considering the survey nature of the data, the results were reported as frequency (%) for categorical variables and mean (linearized SE) values for continuous variables. Using an iterative Markov chain Monte Carlo method, multiple imputations were applied since nearly 10% of the data were missing. Imputation was performed for each survey, separately. Supplementary table 1 shows the extent of missing data per variable per survey.

Survey analysis was applied in Stata ver. 14, and the results of all STEPS surveys were weighted according to the national census categories of the province, residential area (rural, urban), sex, and age (40 – <50 years, ≥50 years). The size of the population for these categories was extracted from the national censuses of 2006, 2011, and 2016 accordingly. Furthermore, to standardize the results, a ‘poststrata’ weight was defined based on sex and age categories of the national Iranian census of 2011 as a reference population.

The trend of CVD risk, and also the risk factors, was examined using generalized linear models including linear regression for continuous outcomes and logistic regression for binary outcomes. CVD risk or CVD risk factors were considered the dependent variable (outcome) and the time of the survey was the independent variable. Marginal means were reported for each time and the linear trend was assessed based on the coefficient of the time.

WHO cardiovascular disease risks were estimated using non-laboratory and laboratory-based approaches. We carried out the analysis for men and women and diabetic and non-diabetic individuals separately. The interaction between the trend of CVD risk and sex or diabetes status was evaluated by adding the interaction term of time × sex/diabetes to the models in the pooled data.

Since the non-laboratory model is used as the first step of a screening program, and high-risk people are referred for more evaluation using lab measurements, we assessed the performance of the no-lab model assuming the laboratory model as the gold standard. To do this, we considered the risk threshold of 20% for defining high-risk people based on the models because it is a known risk threshold for intensive care including pharmacological intervention in high-risk individuals [10]. The traditional indices of performance including sensitivity, specificity, and predictive values were measured. To increase the sensitivity of the non-laboratory model we also considered the risk threshold of 10% for the non-laboratory model to detect high-risk individuals considering the lab-based risk threshold of 20%.

## Results

In all 62,076 (31,660 women) participants aged 40–65 years were included in this study. Table 1 depicts the distribution of CVD risk factors throughout the studies, in men and women, separately. As shown, the mean age was the same in men and women for different years. Overall, compared to men, the mean values of BMI were higher in women [29.2 kg/m^2^ (95%CI: 29.1–29.3) in women and 26.4 kg/m^2^ (26.3–26.6) in men, in 2016, P < 0.001]. However, the trend was increasing in men (from 25.9 kg/m^2^ in 2007 to 26.4 kg/m^2^ in 2016, P < 0.001). The mean values of SBP significantly decreased during recent years in men (from 129.7 mm Hg to 127.7 mm Hg. P < 0.001) but fluctuated in women. As expected, a higher prevalence of smoking was detected in men and the highest prevalence was in 2007 (35.9%, 34.2–37.7) with a declining trend. Over the years, the levels of both total cholesterol and HDL-cholesterol have been higher in women than men; in both sexes, the trends were decreasing. The prevalence of diabetes increased from 2007 to 2011 but considerably decreased from 2011 to 2016 (Table 1).

**Table 1:** The distribution of CVD risk factors in the Iranian general population aged 40–65 years, 2005–2016.


	2007	2008	2009	2011	2016

**Men**					

**Number**	7,519	7,486	7,269	1,802	6,340

**Age (year)**	50.5 (50.4–50.6)	50.5 (50.3–50.6)	50.7 (50.6–50.8)	50.8 (50.6–51.0)	50.2 (50.1–50.3)

**BMI (kg/m^2^)**	25.9 (25.8–26.1)	25.7 (25.6–25.8)	25.7 (25.5–25.8)	26.3 (26.0–26.6)	26.5 (26.4–26.6)

**SBP (mmHg)**	129.7 (129.1–130.3)	128.1 (127.5–128.7)	127.9 (127.3–128.4)	128.9 (128.0–129.8)	127.7 (127.3–128.1)

**DBP (mmHg)**	82.3 (81.9–82.7)	82.2 (81.7–82.6)	82.1 (81.7–82.5)	80.9 (80.3–81.5)	80.2 (79.9–80.5)

**Current smoking**	35.9 (34.2–37.7)	34.7 (33.0–34.6)	34.8 (33.1–36.6)	33.2 (30.5–35.9)	30.0 (28.8–31.1)

**Total Cholesterol (mg/dl)**	189.9 (188.3–191.4)	–	–	180.9 (178.5–183.2)	164.5 (163.6–165.4)

**HDL_Cholesterol (mg/dl)**	41.1 (40.6–41.6)	–	–	42.2 (41.7–42.8)	37.6 (37.4–37.9)

**Fasting Plasma Glucose (mg/dl)**	98.0 (96.4–99.6)	–	–	102.8 (100.7–105.0)	101.1 (100.2–102.0)

**Diabetes**	15.1 (13.7–16.5)	–	–	20.9 (18.8–23.1)	16.3 (15.4–17.2)

**Women**					

**Number**	7,481	7,417	7,187	2,608	6,967

**Age (year)**	50.7 (50.6–50.8)	50.6 (50.5–50.7)	50.9 (50.7–51.0)	50.9 (50.8–51.1)	50.4 (50.4–50.5)

**BMI (kg/m^2^)**	29.0 (28.8–29.1)	28.4 (28.3–28.6)	28.6 (28.4–28.7)	26.7 (28.4–28.9)	29.2 (29.1–29.3)

**SBP (mmHg)**	129.4 (128.7–130.1)	127.4 (126.7–128.1)	127.3 (126.7–128.0)	129.0 (128.2–129.8)	129.5 (129.1–130.0)

**DBP (mmHg)**	84.4 (84.0–84.8)	83.5 (83.1–83.9)	83.4 (82.9– 83.8)	82.1 (81.5–82.6)	79.5 (79.2–79.8)

**Current smoking**	5.3 (4.7–6.1)	4.6 (3.9–5.3)	5.2 (4.4–6.0)	4.3 (3.6–5.1)	4.4 (3.9–4.9)

**Total Cholesterol (mg/dl)**	203.2 (201.7–204.7)	–	–	197.0 (195.2–198.8)	173.1 (172.2–173.9)

**HDL_Cholesterol (mg/dl)**	44.7 (44.3–45.1)	–	–	47.4 (46.9–47.9)	43.5 (43.2–43.8)

**Fasting Plasma Glucose (mg/dl)**	98.9 (97.6–100.1)	–	–	107.1 (105.3–109.0)	104.2 (103.3–105.1)

**Diabetes**	15.4 (14.2–16.7)	–	–	24.2 (22.5–26.0)	19.2 (18.3–20.1)


All p-for trends of CVD risk factors are <0.05.

**Figure 1:**
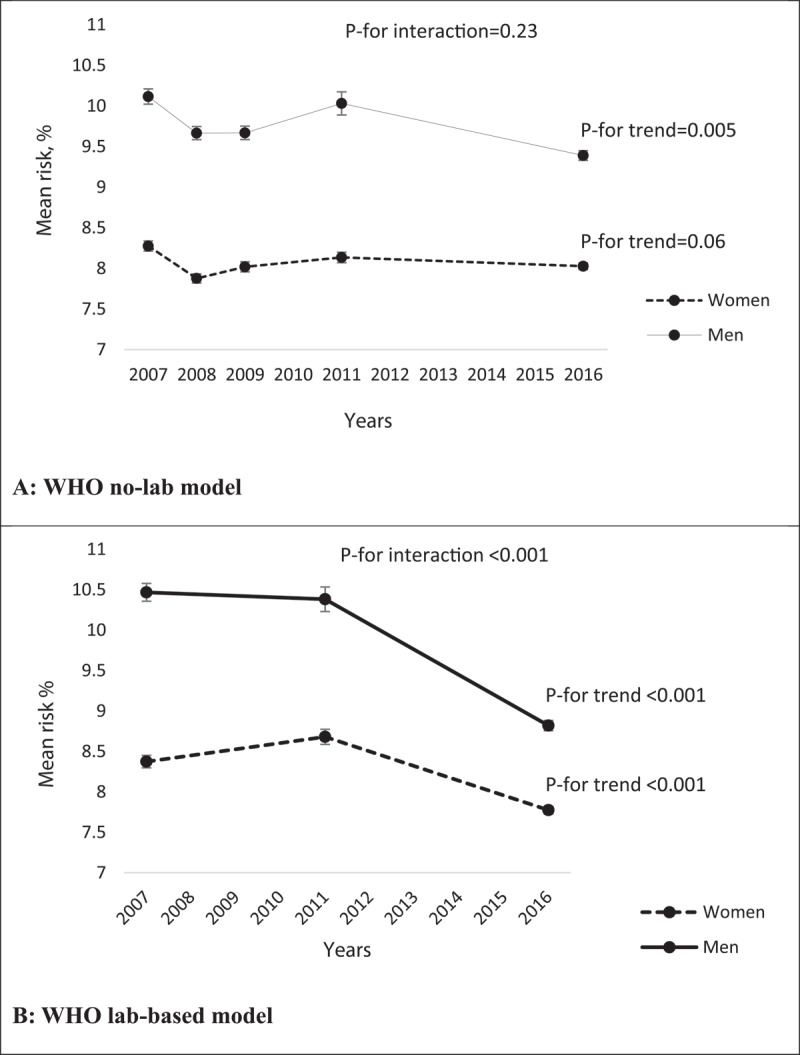
The trend of mean CVD risk in men and women: 2007–2016. A: WHO no-lab model. B: WHO lab-based model.

The trend of the mean CVD risk, as a summary measure of all risk factors, between 2007 and 2016 is presented in Figure 1 for men and women, separately. As shown, significant declining trends were detected for the mean CVD risk in men in both laboratory and non-laboratory models (from 10.5% to 8.8%, P-for trend <0.001 in the laboratory and 10.1% to 9.4%, P-for trend <0.005 in the non-laboratory model, 2007 vs. 2016). In women, the trend was not statistically significant in the no-lab-based model (P-for trend = 0.06) while a significant reduction was observed based on the laboratory model (from 8.4% in 2007 to 7.8% in 2016, P-for trend <0.001). The laboratory model showed a greater decrease in men than women (P-for interaction <0.001).

Table 2 shows the CVD risk categories in the study population. In men, considering the non-laboratory model, 39.3% had a risk ≥of 10% in 2007 while this value decreased to 36.1% in 2016. Considering the laboratory model, this decrease was from 40% to 31.5%. Among women, the non-laboratory model showed a decrease from 29.5% to 28.2% for the risk category of ≥10% from 2007 to 2016. The corresponding values for the laboratory model were 29.8% to 26.1%.

**Table 2:** The proportion of CVD risk levels in the Iranian general population aged 40–65 years during 2007–2016.


	2007	2008	2009	2011	2016

**Men**					

**WHO non-laboratory model**					

**Risk <10%**	60.7 (59.3–62.0)	61.7 (60.2–63.1)	61.9 (60.6–63.3)	59.8 (57.8–61.8)	63.8 (62.8–64.8)

**10 ≤ Risk <20**	30.5 (29.1–31.2)	31.1(29.8–32.7)	31.5 (30.1–32.9)	31.9 (30.0–33.9)	29.7 (28.7–30.7)

**20 ≤ Risk <30**	7.2 (6.5–8.1)	6.0 (5.3–6.7)	5.6 (4.9–6.3)	6.7 (5.7–7.8)	5.5 (5.0–6.1)

**30 ≤ Risk**	1.6 (1.2–2.1)	1.1 (0.8–1.5)	0.9 (0.6–1.4)	1.6 (1.0–2.4)	0.9 (0.7–1.2)

**WHO laboratory model**					

**Risk <10%**	60.0 (58.6–61.4)	–	–	59.8 (57.6–61.9)	68.5 (67.5–69.4)

**10 ≤ Risk <20**	29.6 (28.1–31.1)	–	–	29.6 (27.6–31.8)	25.0 (24.1–26.0)

**20 ≤ Risk <30**	8.0 (7.2–8.9)	–	–	8.0 (6.9–9.2)	5.3 (4.8–5.8)

**30 ≤ Risk**	2.4 (1.9–2.9)	–	–	2.5 (2.0–3.3)	1.2 (0.1–1.4)

**Women**					

**WHO non-laboratory model**					

**Risk <10%**	70.6 (69.4–71.7)	73.6 (72.4–74.7)	71.8 (70.6–73.0)	70.4 (69.1–71.7)	71.8 (71.0–72.6)

**10 ≤ Risk <20**	25.9 (24.7–27.0)	24.1 (23.0–25.3)	25.9 (24.7–27.1)	27.1 (25.8–28.5)	26.0 (21.2–26.8)

**20 ≤ Risk <30**	3.3 (2.8–3.9)	2.2 (1.8–2.6)	2.2 (1.7–2.8)	2.4 (1.9–2.9)	2.1 (1.8–2.5)

**30 ≤ Risk**	0.3 (0.1–0.5)	0.1 (0.07–0.2)	0.1 (0.05–0.2)	0.1 (0.04–0.3)	0.06 (0.03–0.1)

**WHO laboratory model**					

**Risk <10%**	70.2 (69.0–71.4)	–	–	68.3 (66.8–69.8)	73.8 (72.9–74.7)

**10 ≤ Risk <20**	24.5 (23.4–25.7)	–	–	26.3 (24.8–27.8)	22.1 (21.3–23.0)

**20 ≤ Risk <30**	4.3 (3.8–5.0)	–	–	4.7 (4.0–5.5)	3.7 (3.3–4.1)

**30 ≤ Risk**	1.0 (0.7–1.2)	–	–	0.7 (0.5–1.1)	0.3 (0.2–0.5)


WHO: World Health Organization. For each risk category, the proportion and 95% CI were reported.

Assuming the laboratory model as the gold standard, the performance of the no-lab model is presented in Table 3. Compared to the women, the non-laboratory model revealed a higher sensitivity [87.2% (86.4–88.0)] and positive predictive value [83.8% (82.9–84.6)] in men at the risk threshold of 10%. To use the non-laboratory model as the first step of a screening program, we re-assessed the sensitivity, specificity, and predictive values of the non-laboratory model in the risk threshold of 10% to detect high-risk individuals considering the lab-based risk threshold of 20%. In this case, the non-laboratory model has high sensitivity and negative predictive values of 99.4% and 99.9% in women and 98.2% and 99.6% in men, respectively.

**Table 3:** The clinical performance of the WHO non-laboratory model in the Iranian general population aged 40–65 years.


	MEN (N = 15,661)	WOMEN (N = 17,056)

**WHO non-laboratory model**		

**Sensitivity, % (95%CI)^a^**	87.2 (86.4–88.0)	82.7 (81.8–83.7)

**Specificity, % (95%CI)^a^**	87.0 (86.3–87.7)	89.9 (89.3–90.4)

**PPV, % (95%CI)^a^**	83.8 (82.9–84.6)	80.6 (79.5–81.6)

**NPV, % (95%CI)^a^**	89.9 (89.2–90.5)	91.1 (90.6–91.7)

**Sensitivity, % (95%CI)^b^**	98.1 (97.4–98.7)	99.4 (98.7–99.8)

**Specificity, % (95%CI)^b^**	61.2 (60.4–62.0)	69.4 (68.7–70.2)

**PPV, % (95%CI)^b^**	23.5 (22.5–24.5)	16.6 (15.7–17.6)

**NPV, % (95%CI)^b^**	99.6 (99.5–99.7)	99.9 (99.9–100.0)


PPV: positive predictive value; NPV: negative predictive value; WHO: world health organization. ^a^ Model performance was estimated considering the cut-off point of 20% for defining high-risk individuals in both laboratory and non-laboratory models. ^b^ Model performance was estimated considering the cut-off point of 20% and 10% for defining high-risk individuals in the laboratory and non-laboratory models, respectively.

**Figure 2:**
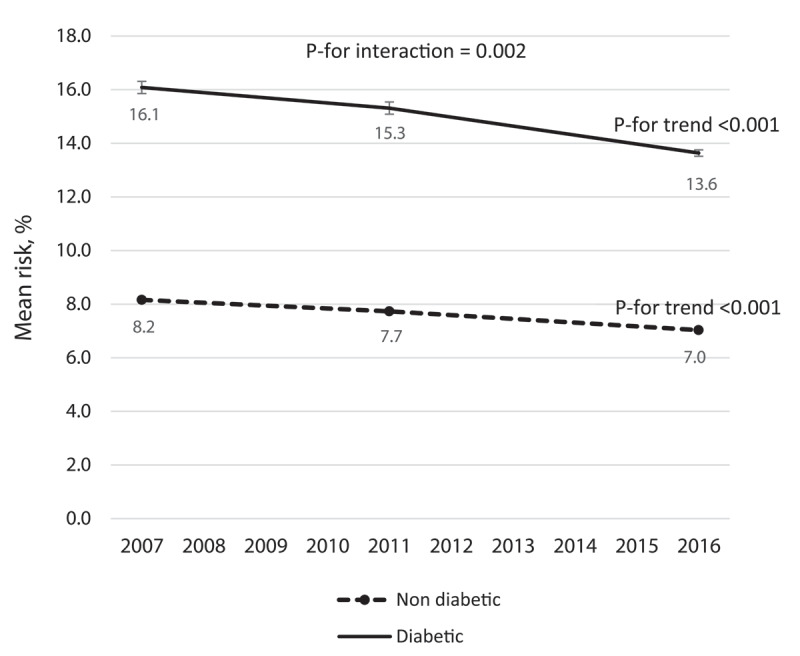
The trend of mean CVD riskin those with and without diabetes between 2007 and 2016.

The trends of laboratory WHO CVD risk among diabetic and non-diabetic participants are presented in Figure 2. As shown, the mean risk was decreased in both diabetic (from 16.1% to 13.6%, P-for trend < 0.001) and non-diabetic (from 8.2% to 7%, P-for trend < 0.001) individuals, but individuals with diabetes experienced greater reductions compared to the non-diabetic participants (P-for interaction = 0.002).

## Discussion

The purpose of this study was to use national data from STEPS studies in the last decade to investigate the 10-year (2007–2016) time trend of the Iranian population’s CVD risk. The WHO laboratory and non-laboratory models were used to analyze trends in the mean CVD risk in men and women and also in diabetic and non-diabetic populations. We showed that the trends of different CVD risk factors are different but the trend of CVD risk score is generally decreasing; this decrease was more evident in men and also in diabetic population.

CVD risk screening is a recognized primary prevention strategy by calculating a risk score that combines known cardiovascular risk factors. The WHO and ACC/AHA guidelines for primary prevention of CVD in public and clinical health settings recommend using a CVD risk score assessment to identify at-risk individuals and determine the appropriate intensity of lifestyle and preventive pharmacological interventions [4,9]. Previous studies have shown that lifestyle interventions combined with or without pharmacotherapy can significantly reduce CVD risk scores [11]. Furthermore, risk factors usually complement each other. Most cardiovascular events occur in people with a moderate increase in several risk factors rather than in people with a significant increase in one of the individual risk factors. So, it is not cost-effective to look at one of the risk factors for cardiovascular diseases alone to identify high-risk individuals. A more cost-effective way is to make decisions based on each individual’s risk in predictable future cardiovascular events [12,13].

There have been various assessments of the trend of cardiovascular disease risk factors in Iran and worldwide. Still, studies on the CVD risk score’s trend as a criterion that includes all risk factors for cardiovascular disease have been limited. We reported that the lipid profile status in our population has improved. The smoking rate also has shown a favorable downward movement. However, favorable changes were not observed in some other components of CVD risk scoring, including BMI and diabetes. These results are in line with national and sub-national studies [14,15,16].

Previous studies of the Iranian population have shown that the prevalence of smoking has declined [17]. Our research also agrees with this finding in men and women. Analogous results from other global studies on reducing smoking rates suggest that population-based government programs such as education about the dangers of tobacco and restrictions on cigarette advertisements have been effective [18,19]. Iran has also made the same efforts to control tobacco use in the past decade [20].

The WHO’s laboratory-based risk score showed a downward trend that seems more prominent compared to that in the non-laboratory model in both men and women. This result may be related to the nature of the variables used in the risk score models, mainly total cholesterol which has been replaced by BMI in the non-laboratory model. The ameliorations in CVD risk might attributable to some national strategies.

Iran has an NCDs roadmap according to the WHO best-buys recommendations including policies for reducing sugar, fat, and salt [21]. As in the rest of the world, studies in the Iranian population’s blood lipid profiles have reported a remarkable improvement in the past decade. Health policymakers in Iran designed a national plan and agenda to reduce the amount of Trans Fatty Acids in the oil industry and also focus on public education regarding the adverse health effects of TFA [22], Some studies showed that the levels of TC and non-HDL-C decreased, and the level of HDL-C increased [23]. Favorable changes in the Iranian lipid profiles come from lifestyle modifications and measures that have been executed in the last decades. The use of lipid-lowering drugs has increased dramatically among men and women populations [13], and people have consumed less hydrogenated oil compared to the past [24]. Studies also have shown that smoking affects the blood lipid profile [25]; thus, proposing a reduction of tobacco smoking as a probable approach to improve lipid profile, which is also found in our study population. We also observed more favorable changes in men’s CVD risk scores than women’s. This issue might be due to more prominent improvements in systolic blood pressure and smoking in men than women [15,16].

Apart from the fact that diabetes itself is one of the leading risk factors for the events of CVD [26], a separate assessment of CVD risk scores of diabetic and non-diabetic participants helps us better understand the outcomes of the measures taken in primary and secondary prevention programs in the Iranian population. We found that risk scores had a favorable trend in both diabetic and non-diabetic individuals with a more prominent decrease in diabetic patients which may indicate better performance of health care in secondary prevention compared to the primary level. These results are in parallel with another study on STEPS data which showed a greater declining trend of CVD risk factors levels among the diabetic population [16]. Additionally, a study on the trends of CVD risk factors in diabetic and non-diabetic populations of the TLGS cohort showed that diabetic participants generally had better control of their CVD risk factors than non-diabetic participants [14]. To reduce the burden of diabetes, the national program for prevention and control of diabetes has been developed from 2004 onwards. This program has made great improvement in the sustained care for diabetic patients [27].

The validity of the WHO non-laboratory was compared with the laboratory model in our population, and the non-laboratory risk score assessment showed acceptable sensitivity and specificity. Various attempts have been made to investigate the validities of different non-laboratory models. In 2018, Joseph and colleagues conducted a study in multiple world regions to compare laboratory and non-laboratory INTERHEART CVD risk scores. In their evaluations, non-laboratory risk scores were able to show similar precision to laboratory models [28]. Another study assessing a non-laboratory risk scoring model in the NHANES III population also suggested their non-laboratory CVD risk scoring as a useful alternative risk scoring model for laboratory methods [29]. These findings altogether reveal that the non-laboratory is not inferior to the laboratory model and can be used as a practical risk assessment in low- and middle-income countries that cannot provide laboratory assessment. Our results showed that if we use the non-laboratory model at the risk threshold of 10%, as the first step of a screening program, we would detect almost all individuals with a risk >= 20% in the laboratory model. In this case, based on the 2016 risk distribution, only 36% of men and 28% of women with non-laboratory risk >= 10% need lab measures, and finally, around 6% of men and 4% of women with laboratory risk >= 20% need more intensive intervention like statin therapy besides lifestyle modification. The shortcoming of this strategy may be losing the opportunity to find unknown diabetic patients using blood sugar measurements. Applying a non-laboratory prediction model for screening high-risk people for diabetes, besides the risk scoring for CVD, can compensate for this limitation. Appropriate non-laboratory prediction models for diabetes have been previously validated for the Iranian population [30,31,32]. To end with, although in this study, we preliminary assessed the performance of the non-laboratory model, the validity of the no-lab and lab-based models was not our main objective and we did not go into this issue in detail. Meanwhile, we emphasized that using a non-laboratory model can reduce the cost of laboratory measurements for a large number of the population who are very low risk. On the other hand, we proposed that high-risk individuals based on the non-laboratory model, won’t be considered for managing their high CVD risk unless the risk is confirmed based on the lab-based score.

As a strength, several national population-based surveys for NCD risk factors with large sample sizes led us to high precision in estimating the CVD risk and its trend during the last decade. All surveys used the WHO standard protocols and had a large representative sample. However, some minor differences were between sampling methods which were addressed using post-stratification weighting on age and sex categories of the population in 2011 as a reference for all years. Since the WHO risk scoring is for ages 40–80 years and STEPS data are available for ages 25–65, our analysis was limited to the 40–65 years old population.

To conclude, despite favorable changes in CVD risk, as a surrogate of all risk factors, during the last decade, still, around one-third of our target population is at high risk with a CVD risk above 10%. The WHO non-laboratory risk score could help detect high-risk people at a low cost. Primary prevention using a stepwise screening for cardiometabolic risk factors using non-laboratory and laboratory prediction models and reducing risk in the general population according to their risk level and appropriate intervention is recommended.

## Data Accessibility Statement

The datasets used during the current study are available from the National Institute of Health Researches of Iran on reasonable request.

## Additional File

The additional file for this article can be found as follows:

10.5334/gh.1180.s1 Supplementary Table 1. The number of missing values per variable in STEPs surveys (2007–2016), among individuals aged 40–65 years included in the current study.
