# Supplementary material for: The Trend of Risk for Cardiovascular Diseases During the Past Decade in Iran, Applying No-Lab and Lab-Based Prediction Models
Source: Glob Heart. 2023 Feb 10;18(1):3. doi: 10.5334/gh.1180 (PMC9951641; doi:10.5334/gh.1180)
Supplement: Supplementary Table 1. — The number of missing values per variable in STEPs surveys (2007–2016), among individuals aged 40–65 years included in the current study. [file gh-18-1-1180-s1.pdf]

**Supplementary Table1**: The number of missing values per variable in STEPs surveys (2007-2016), among individuals aged 40-65 years included in the current study.

| | **2007**<br>**N=15000** | **2008**<br>**N=14903** | **2009**<br>**N=14456** | **2011**<br>**N=4410** | **2016**<br>**N=13307** |
|---|---|---|---|---|---|
| **Age (year)** | 0 | 0 | 0 | 0 | 0 |
| **BMI (kg/m$^2$)** | 0 | 0 | 0 | 47 | 400 |
| **SBP (mmHg)** | 7 | 2 | 5 | 18 | 260 |
| **DBP (mmHg)** | 7 | 13 | 18 | 20 | 264 |
| **Current smoking** | 6 | 3 | 3 | 11 | 225 |
| **Total Cholesterol (mg/dl)** | 2794 | - | - | 1470 | 3476 |
| **HDL_Cholesterol (mg/dl)** | 2776 | - | - | 1470 | 3480 |
| **Fasting Plasma Glucose (mg/dl)** | 2742 | - | - | 1469 | 3546 |
